# A Comprehensive Analysis of Class I Medical Device Recalls: Unveiling Patterns, Causes and Global Impacts

**DOI:** 10.7759/cureus.67542

**Published:** 2024-08-22

**Authors:** Aaliya Parvin M.J., Sudheer Kumar T, Kamaraj R

**Affiliations:** 1 Pharmacy, SRM Institute of Science and Technology, Kattankulathur, IND

**Keywords:** device, usfda, safety, enforcement actions, recall

## Abstract

In the landscape of medical device regulation, Class I recalls serve as pivotal indicators of potential risks, necessitating comprehensive analysis to unveil underlying patterns and causal factors. This research offers a detailed examination of Class 1 recalls, focusing on the critical aspects of device classification, review panel involvement, and the geographic distribution of recalling companies.

Utilizing a robust dataset spanning multiple jurisdictions and device categories, this study reveals recurring trends in recall occurrences, providing insights into the regulatory mechanisms governing device safety assessments. Furthermore, it investigates the role of review panels in evaluating device safety and effectiveness, shedding light on their significance in the recall process.

Moreover, the analysis explores the countries of origin of companies initiating recalls, offering insights into the global impact of regulatory actions on medical device manufacturers. By understanding the drivers behind recall decisions and their implications on a regional scale, regulatory authorities, and healthcare stakeholders can implement targeted measures to enhance patient safety and strengthen post-market surveillance practices.

## Introduction

In the landscape of modern healthcare, medical devices serve as indispensable tools, offering innovative solutions for diagnosis, treatment, and patient care [1]. However, the proliferation of these devices necessitates robust regulatory oversight to ensure their safety, efficacy, and reliability [2]. In this context, the Food and Drug Administration (FDA) in the United States is essential, in supervising the creation, authorization, and post-approval of medical devices [3]. Despite stringent regulations, the rising trend in medical device recalls underscores significant concerns about device safety and regulatory effectiveness. Existing research highlights that device recalls are influenced by manufacturing defects, software issues, and non-compliance with FDA standards. Studies emphasize the need for enhanced quality control and regulatory measures to address these challenges effectively. This research article aims to comprehensively explore medical device regulation, encompassing classification, recalls, and associated procedures as mandated by the US FDA [4].

US FDA

The United States Food and Drug Administration (FDA) is a federal agency within the Department of Health and Human Services (HHS) [5]. The FDA was founded in 1906 to safeguard and advance public health via the regulation of the efficacy and safety of medications, food, pharmaceuticals, biologics, medical devices, cosmetics, and tobacco products. It oversees the entire lifecycle of these products, from premarket approval to post-market surveillance. The FDA is responsible for reviewing and approving new products, ensuring compliance with regulatory standards, conducting inspections, and taking enforcement actions when necessary to safeguard public health [6].

The regulation of medical devices by the US FDA is overseen primarily by the Center for Devices and Radiological Health (CDRH). The CDRH is in charge of ensuring the effectiveness, safety, and quality of medical devices sold in the US. A detailed overview of the regulatory process for medical devices [7].

Classification: Class I, II, and III are the three categories into which medical devices are divided according to the degree of risk that they present to users and patients. Devices in Class I are low-risk, while those in Class III are high-risk [8].

The US FDA's regulation of medical devices aims to balance innovation and patient safety by ensuring that devices are safe, effective, and of high quality. The regulatory process involves rigorous review and evaluation of medical devices to minimize risks and protect public health.

Medical device overview

Medical devices encompass a diverse array of products, ranging from simple instruments to complex machinery, designed to diagnose, treat, or prevent medical conditions. The FDA has classified these devices into three categories according to the degree of risk involved [9]:

Class I

Low-risk devices like tongue depressors and bandages. Most are exempt from FDA premarket notification requirements.

Class II

Moderate-risk devices like powered wheelchairs and surgical drapes. They require special controls for safety and effectiveness, often needing a 510(k) premarket notification.

Class III

High-risk devices like pacemakers and coronary stents. These require a Premarket Approval (PMA) demonstrating safety and effectiveness, often with clinical data.

CDRH

The Center for Devices and Radiological Health (CDRH) is one of the main regulatory bodies within the United States Food and Drug Administration (FDA) [10]. It is in charge of ensuring the efficacy and safety of radiation-emitting products and medical devices sold in the US [11]. The CDRH oversees the regulation of a wide range of medical devices [12].

Key Responsibilities of the CDRH

1. CDRH safeguards US medical device safety via 510(k), PMA, and De Novo premarket reviews [13].

2. After the market launch, CDRH monitors medical device safety and performance through adverse event reports, post-market studies, and facility inspections [14].

3. CDRH enforces QSR, ensuring medical device manufacturers follow good practices [15].

4. CDRH guides and standardizes medical device regulations for manufacturers, professionals, and stakeholders [16].

5. CDRH collaborates internationally to harmonize standards and ease medical device market access globally [17].

CDRH and medical device recalls

Medical device recalls are overseen by the CDRH. Despite stringent regulatory scrutiny, instances may arise where medical devices exhibit defects, pose hazards to patients, or fail to meet regulatory standards. In such cases, the FDA may issue recalls to address these concerns, ensuring the prompt removal or correction of affected devices. Recalls can be classified into three categories [18]: 

Class 1 Recall

The most severe type, where there is a significant risk that using the device could lead to serious health problems or even death. Immediate action is crucial to address the issue.

Class 2 Recall

Involves devices that may cause temporary or reversible health problems, or where the risk of serious adverse health consequences is unlikely.

Class 3 Recall

This is the least serious category, typically involving devices that are not likely to cause any adverse health consequences but may violate FDA labeling or manufacturing regulations.

The Center for Devices and Radiological Health (CDRH) oversees a range of medical device recalls aimed at addressing various issues that could impact patient safety. For instance, recalls may involve cardiac implantable devices due to manufacturing defects that could lead to serious malfunctions, posing significant harm to patients relying on these critical devices. Similarly, diagnostic imaging systems might be recalled due to software errors causing incorrect patient diagnosis or treatment, potentially jeopardizing patient care outcomes. Surgical instruments could also be subject to recall if design flaws result in injuries to patients during surgical procedures, highlighting the importance of ensuring devices are safe and effective in clinical settings. These examples illustrate the diverse reasons for recalls within the medical device industry, emphasizing the CDRH's role in safeguarding public health by addressing potential risks associated with device use through timely regulatory actions.

The CDRH works closely with medical device manufacturers to coordinate recall activities, communicate safety information to healthcare professionals and patients, and ensure that corrective actions are implemented effectively to mitigate risks to public health. Additionally, the CDRH publishes information about recalls on its website and guides stakeholders on how to respond to recall notifications and report adverse events associated with medical devices [19].

Types of medical device recall procedures

Medical device recalls can take various forms, including:

Voluntary Recalls

Initiated by manufacturers or distributors upon discovering device defects or safety concerns. FDA oversees and monitors for corrective actions.

FDA-Initiated Recalls

Mandated by the FDA due to identified significant risks with a device. FDA ensures recalls are conducted to protect public health.

Urgent Recalls and Market Withdrawals

Urgent recalls involve immediate device removal to mitigate serious risks. Market withdrawals occur for non-compliant devices with minimal risk. FDA ensures prompt action to safeguard patient safety.

The FDA outlines detailed recall procedures, including notification requirements, communication strategies, and corrective action plans, to facilitate swift and effective recall management.

Benefits of medical device recalls

Medical device recalls, while often seen as negative events due to potential risks to patient safety, can actually offer several benefits:

1. Medical device recalls swiftly address safety concerns, ensuring patient health and adherence to regulatory standards.

2. They enhance transparency and trust by demonstrating proactive risk management practices.

3. Recalls drive improvements in device quality and safety standards.

4. They play a critical role in enhancing overall healthcare efficacy.

5. Recalls contribute to better patient outcomes by maintaining the integrity of medical devices.

Guidelines related to medical device recalls

While the U.S. Food and Drug Administration (FDA) provides specific guidelines for medical device recalls, it also incorporates principles from international standards, particularly those outlined by the International Organization for Standardization (ISO) [20].

ISO 13485:2016 - Medical Devices - Quality Management Systems - Requirements for Regulatory Purposes

Applicable to medical device manufacturers, this standard specifies requirements for quality management systems. While it doesn't specifically address recalls, compliance with ISO 13485 is often required by regulatory authorities, including the FDA, as part of demonstrating good manufacturing practices (GMP) and regulatory compliance.

ISO 14971:2019 - Medical Devices - Application of Risk Management to Medical Devices

This standard offers guidelines for applying risk management procedures to medical devices at every stage of their lifecycle. It includes requirements for identifying, evaluating, and mitigating risks associated with medical device recalls, helping manufacturers assess the severity and likelihood of adverse events, and implementing appropriate corrective actions.

ISO/TR 20416:2020 - Medical Devices - Information to be Supplied by the Manufacturer for the Management of Medical Devices - Guidance on the Application of ISO 14971

This Technical Report provides additional guidance on implementing the principles of ISO 14971, including specific considerations for managing medical device recalls. It offers practical recommendations for manufacturers on integrating risk management into their recall processes to enhance patient safety and regulatory compliance.

ISO 9001:2015 - Quality Management Systems - Requirements

Despite not being specifically related to medical devices, ISO 9001 provides general requirements for quality management systems applicable to all industries, including medical device manufacturing. Compliance with ISO 9001 can help organizations establish robust processes for managing recalls and ensuring product quality and safety.

## Materials and methods

Data for this study were sourced from the US Food and Drug Administration (FDA) public database, focusing on Class I medical device recalls from January 1, 2020, to December 31, 2023. This timeframe was selected to explore recent trends and changes in the frequency and nature of Class I recalls. The data were systematically recorded in an Excel spreadsheet, capturing crucial information such as the type of medical device involved, the recall date, and the recall reason.

Recall reasons were classified to enable a detailed analysis. These categories included risks of injury (e.g., explosions or burns), inaccuracies in results (such as false readings), software issues, manufacturing defects (e.g., cracked bezels), and non-compliance with FDA standards. Categorizing the reasons for recalls aimed to clarify the underlying issues contributing to these events.

The data were analyzed using Microsoft Excel, focusing on several key areas. First, a trend analysis was conducted to examine the annual number of Class I recalls from 2020 to 2023, identifying any changes or patterns over the four years. Second, the recalls were categorized by their classification (Class I, Class II, and Class III) to assess the frequency of each class and its implications for patient safety. Third, the geographic distribution of recalls was analyzed to understand global variations, particularly in the United States, Europe, and Asia.

Further analysis was conducted by a review panel to identify which medical specialties experienced the highest and lowest recall rates. Specialties such as cardiovascular and general hospitals were noted for their significant number of recalls. Finally, the reasons behind the recalls were analyzed to highlight common issues, including manufacturing defects, software malfunctions, and regulatory non-compliance.

Descriptive statistics were employed to summarize the data, using frequency counts and percentages to represent the number of recalls and the proportion of each recall reason and classification. Data visualization tools in Excel, including bar charts, line graphs, and pie charts, were used to effectively illustrate trends, distributions, and comparisons. The analysis aimed to conclude the increasing trend in medical device recalls the predominance of specific recall classes, and their overall impact on patient safety. This approach highlights the need for enhanced quality control, regulatory oversight, and international cooperation among healthcare providers and manufacturers.

## Results

**Figure 1:**
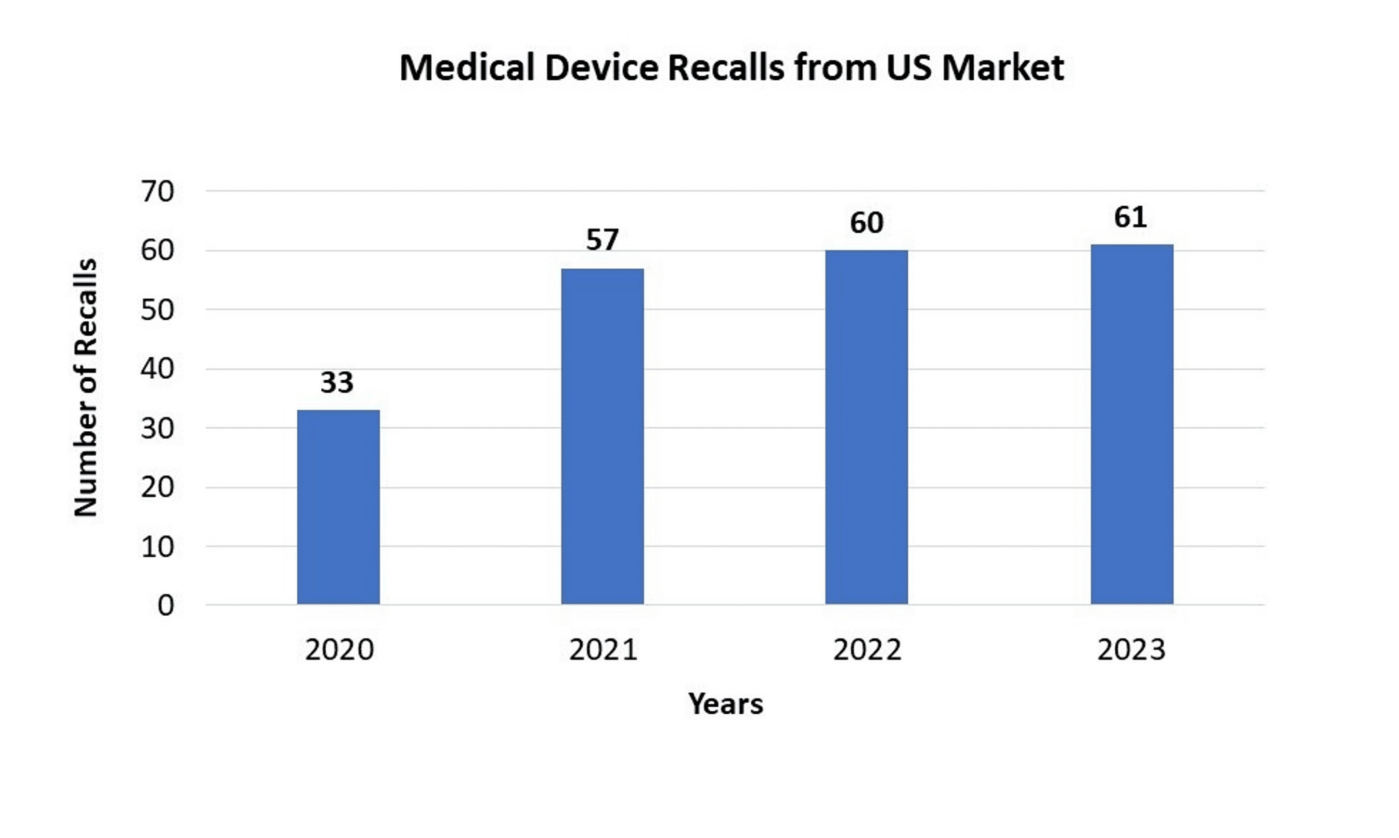
Medical Device Recalls from US Market

Over the course of four years from 2020 to 2023, the US market has witnessed a consistent increase in medical device recalls. The numbers have climbed from 33 in 2020 to 61 in 2023, indicating a growing concern regarding the safety and quality of these products. This upward trend emphasizes the necessity for heightened quality control measures and more stringent regulatory oversight within the US medical device industry. It is crucial for regulatory bodies, manufacturers, and healthcare providers to collaborate closely to address these challenges and prioritize the safety of patients. Healthcare providers must also remain vigilant and informed about these recalls to mitigate potential disruptions in patient care. As the figures continue to rise, maintaining a focus on patient safety and product quality becomes increasingly critical for all stakeholders involved in the US medical device market (Figure 1).

From 2020 to 2023, Class II medical device recalls consistently outnumbered Class I and Class III recalls, highlighting devices with moderate risk. The data reveals 2 Class I recalls, 27 Class II recalls, 4 Class III recalls, and no unclassified devices in 2020. In 2021, this rose slightly to 3 Class I, 37 Class II, 13 Class III, and 4 unclassified. Figures for 2022 fluctuated with 3 Class I, 39 Class II, 6 Class III, and 12 unclassified. In 2023, there were 6 Class I, 47 Class II, 6 Class III, and 2 unclassified recalls.

The dominance of Class II recalls raises concerns about device safety and efficacy. Class III recalls, representing the highest-risk devices, fluctuated but remained significant for patient safety. This data emphasizes the need for robust quality control and regulatory oversight, particularly for Class II and Class III devices. Manufacturers should adhere to strict quality standards to prevent faulty devices from entering the market, and swift, effective recalls are crucial for patient safety.

**Figure 2:**
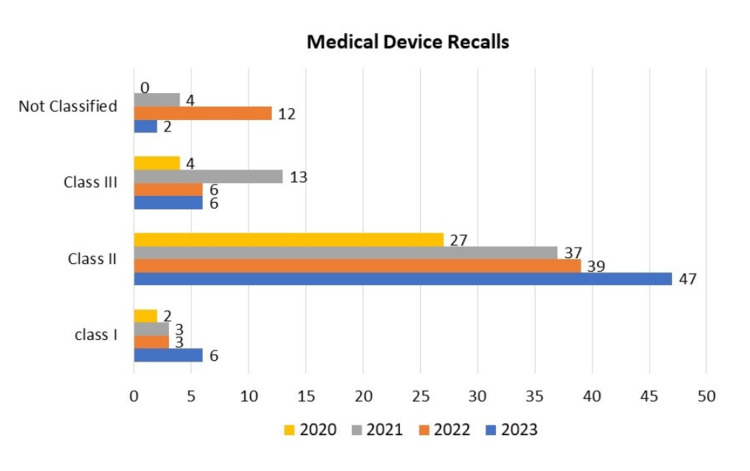
Medical Device Recalls by Classification (2020-2023)

Continued collaboration between healthcare providers and regulatory bodies is vital to minimize risks associated with medical devices. Targeted interventions may be needed for Class II devices, including enhanced monitoring and communication strategies. As the data reveals these trends, a focus on improving safety protocols and communication among stakeholders remains paramount in the medical device industry (Figure 2).

**Figure 3:**
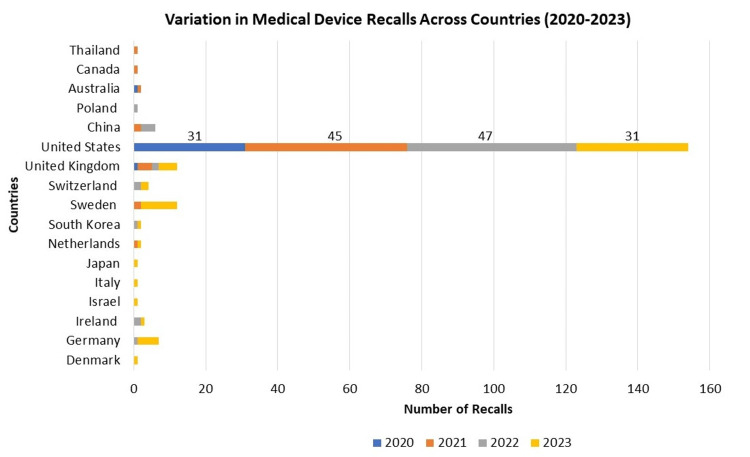
Variation in Medical Device Recalls Across Countries (2020-2023)

From 2020 to 2023, medical device recalls varied across countries. The United States consistently reported the highest numbers, with recalls numbering 31 in 2020, 45 in 2021, and 31 in 2023. European countries like Germany, Sweden, Switzerland, and the United Kingdom also had recalls, with Sweden peaking at 10 in 2023. Asian countries such as Japan, South Korea, and China reported fewer recalls. These differences in recall numbers may reflect each country's unique regulatory landscape and market dynamics. The data emphasizes the necessity for robust monitoring systems globally to ensure device safety. Collaboration among countries is vital for sharing best practices and improving overall device safety standards. Manufacturers must adhere to diverse regulatory requirements across borders to maintain consistent quality control. This data highlights the global nature of medical device recalls and the importance of international cooperation to enhance patient safety and device effectiveness (Figure 3).

**Figure 4:**
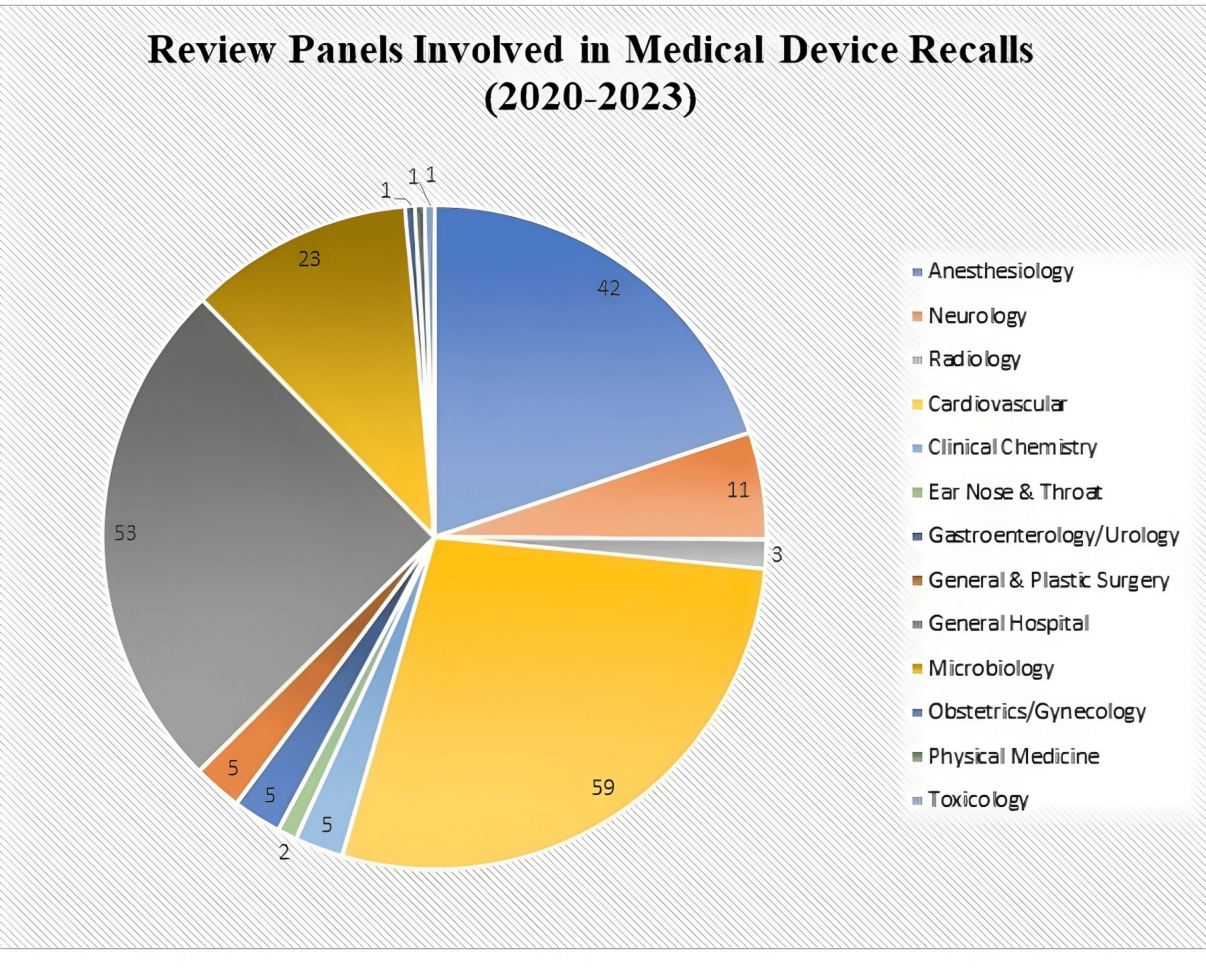
Review Panels Involved in Medical Device Recalls (2020-2023)

The data from 2020 to 2023 (Figure 4) reveals the distribution of medical device recalls by review panel. Cardiovascular procedures had the highest number of recalls with 59 incidents, highlighting critical concerns for heart health and patient safety. General hospital devices and anesthesiology also showed significant recalls, with 53 and 42 incidents respectively, underlining the need for stringent quality control in these areas. Microbiology devices, with 23 recalls, stand out as an area of concern for diagnostic accuracy and patient treatment. Conversely, there were notably low recall rates in specialties like obstetrics/gynecology, physical medicine, toxicology, and radiology. These findings emphasize the critical importance of rigorous testing, monitoring, and continuous evaluation of device safety post-market. Manufacturers and regulatory bodies must focus on enhancing quality control measures, particularly in high-incidence areas like cardiovascular and general hospital devices. Healthcare providers also need to stay informed about these recalls to ensure patient safety and the use of devices meeting the highest safety standards. The data underscores the diverse nature of device issues across medical specialties and the ongoing need for collaboration among stakeholders to maintain patient safety and device effectiveness.

The data spanning from 2020 to 2023 (Table 1) offers a comprehensive insight into the various reasons behind medical device recalls during this period. Among the most frequent issues leading to recalls were risks of injury, including potential explosions, smoke, burns, and defects in catheter hubs. These pose significant threats to patient safety and underscore the importance of stringent quality control measures.

**Table 1:** Medical Device Recalls by Reason (2020- 2023)

RECALL REASON DESCRIPTION	Total (n)	n%
Not authorized, cleared, or approved by the FDA	11	5.21%
Risk associated with catheter hub defect	9	4.26%
Risk of inaccurate or false results	9	4.26%
Risk of explosion/smoke/ burn injury to patients	7	3.31%
Unanticipated system shutdowns	7	3.31%
Risk of leaks potentially exposing providers and patients to hazardous/toxic substances	7	3.31%
Software configuration issues/ software malfunction	7	3.31%
Risk of exposure to harmful chemicals/aluminum	7	3.31%
Faulty alarms/ alarm failures	6	2.84%
Risk of cracked or separated bezel repair posts	5	2.36%
Risk of device separation during usage	5	2.36%
Risk of bacterial/ fungal contamination	4	1.89%
Risk of tip damage occurring during use	4	1.89%
Risk of over-infusion and under-infusion	4	1.89%
Lack of sterIlity/ sterilisation procedure	3	1.42%
Risk of errors in medication labeling	3	1.42%
Risk of battery failure	3	1.42%
Risk of keys getting stuck or becoming unresponsive	3	1.42%
Incompatibilities with syringe pumps	2	0.94%
Foreign substances are present in the air pathway	2	0.94%
Unforeseen short battery run times	2	0.94%
Potential for reduced oxygen supply	2	0.94%
Risk of moisture ingress may lead to electrical shorts and shortened battery life	2	0.94%
Potential electrical failure, short circuit	2	0.94%
Risk of breaks and tears during setup process	2	0.94%
Balloon deflation and separation concern	2	0.94%
Risk of device fracture	2	0.94%
Due to an Impaired or Inability to turn the drive	1	0.47%
Failure to detect air in the line	1	0.47%
Due to damage to the connector piece causing unexpected disconnections	1	0.47%
Malfunctions that cause unintended movement of the robotically assisted surgical device	1	0.47%
Blood or heparin leaking back or from syringe	1	0.47%
Potential incorrect indication of completed infusion	1	0.47%
Stolen defective products	1	0.47%
The unit may over-insufflate (inflate) air into the body with no warning or alarm	1	0.47%
Power management printed circuit board assemblies not meeting ventilator standards	1	0.47%
Leaks and shortcircuits	1	0.47%
Inability to exit MRI mode	1	0.47%
Not opening properly	1	0.47%
Failures in gas loss and gas gain lead to operational issues	1	0.47%
Risk of blood clots	1	0.47%
Defibrillation concerns: energy reduction, unintended voltage, or inaccurate readings possible	1	0.47%
Motor damage risk post transcatheter aortic valve replacement (TAVR) stent contact	1	0.47%
Elevated risk of air embolism	1	0.47%
Risk of inadequate energy output in high voltage therapy	1	0.47%
False-negative troponin may delay or miss myocardial infarction diagnosis	1	0.47%
Manifold failure may lead to gas leaks, interrupting neonatal therapy	1	0.47%
Risk of components becoming loose or detached, potentially restricting breathing support	1	0.47%
Risk of potentially delivering inaccurate or insufficient therapy	1	0.47%
Risk of insufficient ventilation and other injuries due to cracked manifolds	1	0.47%
Potential silicone foam adhesion failure and presence of residual PE-PUR foam debris	1	0.47%
Risk of detector fall posing a potential injury to patients	1	0.47%
Challenges leading to therapy delay, interruption, or under-delivery	1	0.47%
Possibility of unintentional extended pump stops due to critical controller failure	1	0.47%
Risk of potential false susceptibility results	1	0.47%
Risk of radiofrequency interference with nearby medical equipment	1	0.47%
Potential risks: battery swelling, leakage, or severe overheating	1	0.47%
Risk: splitting or detaching may cause leakage, impacting air supply	1	0.47%
Safety concerns related to magnets that might impact specific medical devices	1	0.47%
Risk of unintentional extended pump stop in case of controller critical failure	1	0.47%
Risk of airway obstruction	1	0.47%
Risk of inaccurate intracranial pressure readings	1	0.47%
Risk of breathing support interruption due to potential water ingress	1	0.47%
Risk: Short circuit alert and reduced energy shock during therapy	1	0.47%
Issues potentially causing delayed delivery of treatment	1	0.47%
Damaged or fractured power switches in the suction system	1	0.47%
Risk of respiratory distress in ventilated patients during home use	1	0.47%
Defect in the pump weld	1	0.47%
An issue that may lead to ventilator cessation with or without alarms	1	0.47%
Risk of misplaced enteral tubes that could result in patient harm	1	0.47%
Recalls DiaTrust COVID-19 Ag Rapid Test Kits sent to unauthorized users	1	0.47%
Risk of capsule breakage during use	1	0.47%
Distributed to customers without proper training for safe nasopharyngeal swab collection	1	0.47%
Risk of ventilator cessation due to expired adhesive, with or without an alarm	1	0.47%
Hazards associated with PE-PUR foam	1	0.47%
Reports of filter breakage during retrieval	1	0.47%
Potential risk of inaccurate biopsy depth gauge cycle view	1	0.47%
Assembly error during manufacturing	1	0.47%
Defective plunger in prefilled syringe (0.9% sodium chloride)	1	0.47%
Possible cybersecurity vulnerabilities	1	0.47%
Decreased gas flow to patients during anesthesia	1	0.47%
Risk of marker bands moving or dislodging	1	0.47%
Potential for fractures in the delivery system during device placement, retrieval, or movement	1	0.47%
Potential issue with cracked or separated bezel repair posts	1	0.47%
Potential for air re-entering the syringe, leading to an air embolism	1	0.47%
Absence of Instructions for Use for the safety scalpel N11	1	0.47%
Potential for neurological adverse events, mortality, and the risk of failure to restart	1	0.47%
Risk of transition to safety mode	1	0.47%
Potential health hazards associated with PE-PUR sound abatement foam	1	0.47%
Potential for incorrectly low readings	1	0.47%
Inaccurate syringe marks risk over/underdose	1	0.47%
Risk of stent migration poses a potential concern in medical interventions	1	0.47%
Revised details on carrying case, driveline cover, and controller power-up issues	1	0.47%
Risk of controller port damage	1	0.47%
Risk of stent fractures and type III endoleaks	1	0.47%
Risk of fragmented O-ring pieces entering arteries during use	1	0.47%
Potential for broken or bent needles	1	0.47%
Potential delay or failure in restarting after pump cessation	1	0.47%
Failure to correctly secure Q-Link Strap Lock (Q-Link 1 Strap Lock) to S65 hook	1	0.47%
Device damage due to manufacturing error	1	0.47%
Quality problems	1	0.47%
Potential for incorrect display of syringe types and/or sizes	1	0.47%
Damaged connectors, missing battery screws, and broken hinge posts with frame damage	1	0.47%
Risk of medication delivery error	1	0.47%
Partial or complete image loss during operation	1	0.47%
Potential breakdown of motor connector wires	1	0.47%
Risks of Polymer Leaks During Implantation	1	0.47%
Inaccuracies in deep brain stimulation (DBS) procedures	1	0.47%
Detachment of the tip	1	0.47%
Displaying the reversed image	1	0.47%
Fully and partially blocked needles	1	0.47%
Malfunction leading to potential entry of water into the airway	1	0.47%
Incorrect insulin dosage	1	0.47%
Mechanical ventilation loss	1	0.47%
Inaccurate oxygen values	1	0.47%
Valve displacement	1	0.47%

Inaccurate results were also a common cause for concern, with several recalls attributed to the risk of providing false or misleading readings. Software malfunctions were noted as well, highlighting the critical role of software integrity in medical devices. Manufacturing defects, such as cracked or separated bezels and device separations during usage, were also observed, indicating potential weaknesses in the production process.

Patient safety remains paramount in these recall reasons, with notable mentions of risks like bacterial or fungal contamination, blood clots, and the potential for airway obstructions. Operational disruptions, such as unanticipated system shutdowns and risks of keys becoming unresponsive, could pose challenges in healthcare settings where prompt and accurate device operation is crucial.

Non-compliance with FDA standards emerged as a significant issue, as evidenced by recalls for devices not authorized, cleared, or approved by the FDA. This lack of adherence to regulatory standards can have severe implications for patient safety and the overall effectiveness of medical devices. Additionally, cybersecurity vulnerabilities were mentioned, highlighting the increasing importance of data security in medical technology.

The breadth of reasons for recalls underscores the complexity of challenges faced in the medical device industry. It emphasizes the necessity for continuous monitoring, robust quality control protocols, and rigorous regulatory oversight. Healthcare providers must remain vigilant and stay informed about these recall reasons to ensure patient safety and accurate device use. Efforts in these areas are crucial to maintaining the trust and reliability of medical devices in healthcare settings.

## Discussion

The analysis of medical device recalls in the US market over the four years from 2020 to 2023 reveals a significant and concerning trend. The number of recalls steadily increased from 33 in 2020 to 61 in 2023 (Figure 1), indicating a persistent challenge in ensuring the safety and reliability of medical devices available to patients. This upward trajectory underscores the urgent need for strengthened quality control measures and enhanced regulatory oversight within the medical device industry.

Throughout the study period, Class II recalls consistently outnumbered Class I and Class III recalls (Figure 2). Class II recalls, which signify devices with moderate risk levels, were predominant, highlighting ongoing issues with device safety and efficacy across various product categories. The fluctuation in Class III recalls, representing the highest-risk devices, underscores the critical nature of these devices for patient safety and the complexities involved in their design, manufacturing, and post-market surveillance.

Comparing recall patterns internationally (Figure 3), the United States consistently reported the highest number of recalls, followed by select European countries, and fewer incidents were reported in Asian regions. These differences reflect varying regulatory environments and market dynamics, emphasizing the need for global collaboration to establish uniform safety standards and regulatory practices.

Examining recalls by specialty (Figure 4) provides further insights into specific areas of concern. Cardiovascular procedures and general hospital devices experienced the highest number of recalls, indicating significant safety challenges in these critical healthcare domains. Conversely, specialties such as obstetrics/gynecology and radiology reported lower recall rates, suggesting relatively fewer safety issues but highlighting the need for continued vigilance across all medical disciplines.

Root cause analysis (Table 1) identifies a range of issues contributing to device recalls, including risks of injury such as explosions, burns, and device malfunctions. Inaccurate results, software failures, and manufacturing defects were also prevalent, underscoring vulnerabilities in product design, production processes, and regulatory compliance. Non-compliance with FDA standards emerged as a notable issue, highlighting gaps in quality assurance and regulatory adherence that can compromise patient safety and device effectiveness.

The implications of these findings are profound for both industry stakeholders and regulatory bodies. Enhanced quality control measures are imperative to prevent defective devices from entering the market, while robust regulatory oversight is essential to enforce compliance with safety standards and facilitate prompt recall actions when necessary. Effective communication and collaboration among manufacturers, healthcare providers, and regulatory agencies are critical to minimizing risks and ensuring patient safety throughout the device lifecycle.

## Conclusions

The analysis of medical device recalls from 2020 to 2023 highlights a concerning trend of increasing numbers, underscoring the importance of stringent quality control and regulatory oversight. The rise in recalls, particularly in Class II devices, signals potential risks to patient safety and device efficacy. This emphasizes the critical need for manufacturers to adhere to rigorous quality standards to prevent faulty products from entering the market. Collaboration between healthcare providers and regulatory bodies remains essential to mitigate risks and ensure patient safety. The data also sheds light on the global nature of these challenges, with varying recall numbers across countries. Anesthesiology and cardiovascular procedures have shown a notable frequency of recalls, suggesting areas that require particular attention. The distribution of recalls by review panel highlights the diverse range of device issues across medical specialties such as unauthorized devices not cleared by the FDA, catheter hub defects, and risks of inaccurate results. Issues such as explosions, system shutdowns, leaks of hazardous substances, and software malfunctions pose significant safety risks. Manufacturing defects like cracked repair posts and device separations during use also feature prominently. These findings underscore the critical need for improved quality control measures, adherence to FDA standards, and robust regulatory oversight to ensure patient safety and device effectiveness. By prioritizing patient safety and stringent quality standards, we can strive toward a more reliable and safer medical device landscape.
